# Quantitative Analysis of Complement Membrane Attack Complex Proteins Associated with Extracellular Vesicles

**DOI:** 10.3390/proteomes12030021

**Published:** 2024-07-12

**Authors:** Illarion V. Turko

**Affiliations:** Institute for Bioscience and Biotechnology Research, National Institute of Standards and Technology, University of Maryland, Rockville, ML 20850, USA; iturko@umd.edu

**Keywords:** membrane attack complex, extracellular vesicles, multiple reaction monitoring, targeted protein quantification, mass spectrometry

## Abstract

Extracellular vesicles (EVs) represent a universal mechanism of intercellular communication in normal and pathological conditions. There are reports showing the presence of complement proteins in EV preparations, specifically those that can form a membrane attack complex (MAC). In the present work, we have used a quantitative mass spectrometry method that allows for the measurement of multiple targeted proteins in one experimental run. The quantification of MAC-forming proteins, namely C5b, C6, C7, C8, and C9, in highly purified EVs from normal human plasma revealed the presence of MAC proteins at approximately equal stoichiometry that does not fit the expected stoichiometry of preformed MAC. We concluded that while MAC proteins can be associated with EVs from normal plasma and presumably can be delivered to the recipient cells, there is no evidence that the EVs carry preformed MAC.

## 1. Introduction

Extracellular vesicles (EVs) are secreted by all cells and play essential roles in intercellular communication by delivering their cargo of biomolecules to the recipient cells, thereby triggering various physiological and pathological processes. Due to their endogenous ability to deliver messages to the targeted cells, EVs have attracted significant attention as a promising new class of therapeutics with site-specific delivery without off-target side effects [1,2,3,4,5]. To understand EV properties in depth, purified EV preparations need to be obtained. The challenges and approaches associated with EV isolation have been broadly reviewed [6,7]. However, the generation of highly purified EV preparations remains difficult, and analytical techniques to verify EV purity are needed to avoid the misinterpretation of experimental data.

The complement system is a part of the innate immune system and consists of about 50 proteins. These proteins circulate in the blood at a very high abundance and can be commonly found in plasma EV preparations [8,9]. Whether complement proteins simply contaminate EV, preparations because of their high abundance or are functionally associated with EVs remains unclear. One important function of the complement system seems especially relevant to EVs. This is an ability to attack the pathogen’s cell membrane. The complement membrane attack complex (MAC) forms pores in the plasma membrane of pathogens or targeted cells, resulting in their osmolysis [10,11]. MAC assembly includes the soluble complement proteins C5b, C6, C7, C8, and C9, which assemble on the plasma membrane into an asymmetrical split-washer configuration composed of the asymmetric region, a hinge region, and a C9 oligomer [12]. For the C9 oligomer in the MAC, 18 contiguous C9 units have been identified [11]. Therefore, the stoichiometry of MAC is an important consideration when using mass spectrometry to distinguish between irreversibly assembled MAC and the constituent complement proteins C5b, C6, C7, C8, and C9 present in circulation.

There is increasing evidence that EVs can carry complement proteins and that the interplay between EVs and complement contributes to the pro- and anti-inflammatory immune balance affecting the course of disease [9]. The shedding of EVs that carry MAC (MAC-EVs) has been reported as a mechanism of complement resistance under certain disease conditions [13,14]. The outcome of MAC-EVs release is not yet known, but presumably, they can mediate inflammation and neurotoxicity and further contribute to disease progression [15,16,17,18,19]. However, at the present time, the molecular mechanisms behind the EV and complement relationship remain in the earlier stages of research and require selective and quantitative methods to approach them.

In the present work, we propose to use a multiple reaction monitoring (MRM) mass spectrometry assay [20,21], also called selected reaction monitoring (SRM) assay [22], to quantify C5b, C6, C7, C8a, C8b, and C9 complement proteins in various preparations of human plasma EVs. For the purpose of an accurate assignment of the stoichiometry of these proteins in final EV preparations, we used ^15^N-labeled quantitative concatemers (QconCATs) as internal standards [23,24]. This approach allows for the simultaneous quantification of proteins of interest in a single liquid chromatography-mass spectrometry run. To ensure a high level of EV purity, a three-step isolation protocol was used [25]. The efficiency of this protocol was confirmed through the quantification of several EV proteins in parallel with the quantification of abundant plasma proteins, such as albumin and alpha-2-macroglobulin [25]. In the present research, the protocol was used to quantify 13 EV proteins side-by-side with MAC proteins.

## 2. Methods and Materials

### 2.1. Isolation of Plasma EVs

EVs were isolated from 200 mL of pooled normal male human plasma K2EDTA (BioreclamationIVT, Westbury, NY, USA), as described previously [25]. The lot was pooled from 10 donors. This study (MML-16-0062) was initially approved by the NIST Research Protections Office on the 30 April 2021 and undergoes annual report approvals. Briefly, the isolation protocol started with differential centrifugation at 20,000× *g* and 106,000× *g* that generated 20K and 106K EVs, respectively. These two subpopulations were further purified using size-exclusion chromatography on a Superdex 200 Increase 10/300 GL column (GE Healthcare, Chicago, IL, USA) and affinity chromatography on the HiTrap Heparin HP (1 mL) column (GE Healthcare, Chicago, IL, USA) using AKTA FPLC. To reflect the purification steps, the final preparations are called 20K-SEC-Hep EVs and 106K-SEC-Hep EVs, respectively. The total protein in these preparations was measured using a DC protein assay kit (Bio-Rad Laboratories, Hercules, CA, USA).

### 2.2. Dynamic Light Scattering

Dynamic light scattering (DLS) measurements were performed for 20K-SEC-Hep and 106K-SEC-Hep EVs samples using a Malvern Zetasizer Nano series, ZEN3600, and analyzed using Malvern Zetasizer 7.10 software (Malvern Instruments Ltd., Worcestershire, UK). All samples were filtered through a Millipore Millex-GV 0.45 µm PVDF filter and recorded in triplicate at 25 °C.

### 2.3. ^15^N-Labeled Internal Standards for Quantitative Proteomic Analysis

The design, expression, purification, and characterization of ^15^N-labeled EXO1, GP1, GP2, Pl_in,_ and Pl_out_ QconCATs have been previously described in detail [26,27]. These QconCATs were developed for a broad analysis of EV proteins. In addition, two new QconCATs were generated to quantify complement proteins, namely C6, C7, C8a, C8b, and C9 (COM1) and C5a, C5b, C3a, and C3b (COM2). Both COM1 and COM2 QconCATs (Figure S1, Supplemental Material) were expressed as ^15^N-labeled and characterized identically to previously described ones [26,27].

Optimal MRM transitions for Q-peptides were experimentally determined using an MRM assay after tryptic digestion and used to estimate the level of stable isotope incorporation in COM1 and COM2 QconCATs. The values were found to be greater than 99% and accepted as complete labeling. For those proteins for which quantification was included in this study, three optimal MRM transitions per each Q-peptide are shown in Table S1 (Supplementary Material). Since the same Q-peptides are present in some complement proteins and in their fragments, we measured their sum. For example, both peptides used for C3 quantification (IPIEDGSGEVVLSR and VLLDGVQNPR) are present in C3 and C3b; we measured the sum of these proteins (C3/C3b) but called it C3. The same is applicable to C5/C5b, C8/C8a, and C8/C8b.

### 2.4. Sample Processing and MRM Assay

The 20K-SEC-Hep EVs (200 µg of the total protein) and 106K-SEC-Hep EVs (200 µg of the total protein) samples in 50 mmol/L NH_4_HCO_3_ were supplemented with ^15^N-labeled COM1, COM2, EXO1, GP1, GP2, Pl_in_, and Pl_out_ QconCATs (from 1 to 10 pmol each). In a separate set of experiments, to verify the linearity of the MRM assay, several individual amounts of ^15^N-labeled COM1 QconCAT (1 pmol, 2 pmol, 4 pmol, 8 pmol, 16 pmol, and 32 pmol) were spiked into identical aliquots of the 20K-SEC-Hep EV sample. All samples were then treated with 10 mmol/L of dithiotheitol for 60 min and alkylated with 30 mmol/L of iodoacetamide for another 60 min. Trypsin digestion in 50 mmol/L of NH_4_HCO_3_ with 0.1% RapiGest at a 1:5 *w*/*w* ratio (trypsin/protein) was performed as described before [26].

Instrumental analyses were performed on an Agilent 6490 iFunnel Triple Quadrupole LC/MS system (Santa Clara, CA, USA) equipped with an Agilent 1200 HPLC system (Santa Clara, CA, USA) [26]. Liquid chromatography was performed on an Agilent Zorbax Eclipse Plus C18 RRHD column (2.1 mm × 50 mm, 1.8 µm particle), with a flow rate of 200 µL/min. The elution gradient was created using solvent A (0.1% formic acid in water, volume fraction) and solvent B (acetonitrile containing 0.1% formic acid, volume fraction) and includes 4 steps. The initial step was 3% solvent B in solvent A for 3 min, followed by a gradient from 3% to 30% of solvent B in solvent A for 30 min and another gradient from 30% to 50% for 5 min. Finally, the column was re-equilibrated back to 3% solvent B in solvent A [26].

Light and heavy peak area integration and the calculation of the light-to-heavy peak ratios were performed using Skyline 23.1 (University of Washington, Seattle, WA, USA). We measured 3 transitions per peptide as individual measurements, and the peak ratios for these were averaged to yield the peptide ratios that were further used to calculate the concentration of light peptides (unknown) based on the concentration of QconCAT (known). Since we measured 2 peptides per protein in 3 biological replicates, the data are represented as the mean ± SD (*n* = 18).

## 3. Results and Discussion

### 3.1. 20K-SEC-Hep and 106K-SEC-Hep EVs

We targeted the accurate measurement of protein stoichiometry of complement proteins involved in MAC formation, namely, C5b, C6, C7, C8, and C9 in the 20K-SEC-Hep and 106K-SEC-Hep EV preparations. We used a well-established MRM assay [20,21,22,23,24,25,26,27] to quantify these proteins. Nevertheless, we would like to emphasize key aspects of this technique and details of the EV isolation protocol that contribute to the accuracy and confidence of the measurements.

The first important aspect is that selected Q-peptides to quantify targeted proteins were included in the QconCAT with six amino acid flanking residues to ensure equal trypsin digestion rates for the QconCAT and the targeted protein [28]. Furthermore, the selected Q-peptides do not contain cysteines and methionines, which potential oxidation can affect the accuracy of quantification. In addition, to avoid splitting quantification between multiple proteoforms, the selected Q-peptides do not contain any spliced or post-translational modifications reported for targeted proteins in UniProt (https://www.uniprot.org/, assessed on 9 December 2022).

**Figure 1:**
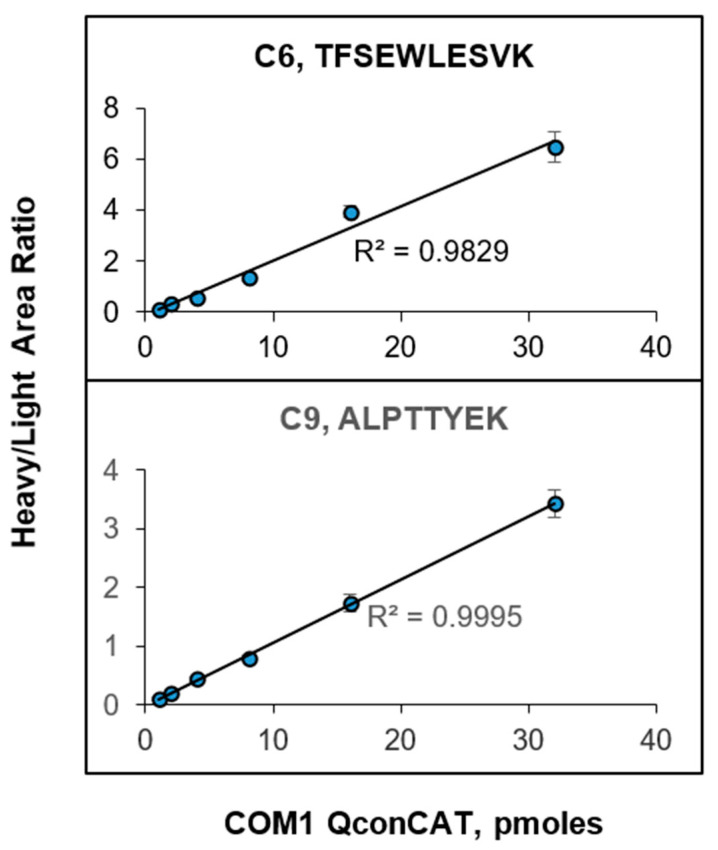
Response curves for 20K-SEC-Hep EVs supplemented with various concentrations of COM1 QconCAT. Two proteins, C6 and C9, were quantified based on a representative peptide. The area ratio of a corresponding heavy peptide to a light peptide (averaged across three transitions and three replicates) was plotted versus COM1 QconCAT concentration. Data are presented as a mean ± SD (*n* = 9).

For linearity experiments (Figure 1), various amounts of COM1 QconCAT were added to the same amount of 20K-SEC-Hep EV samples, and MRM data were plotted as *pmoles* of COM1 QconCAT versus the heavy/light area ratio for representative peptides from C6 and C9. The response curves show linearity and low scatter over the concentration range (from 1 pmol to 32 pmol) of COM1 QconCATs. This is important because it allows for the use of an additional single concentration of ^15^N-labeled QconCATs for targeted protein quantification. It also means consistency in the sample preparation and electrospray conditions.

**Figure 2:**
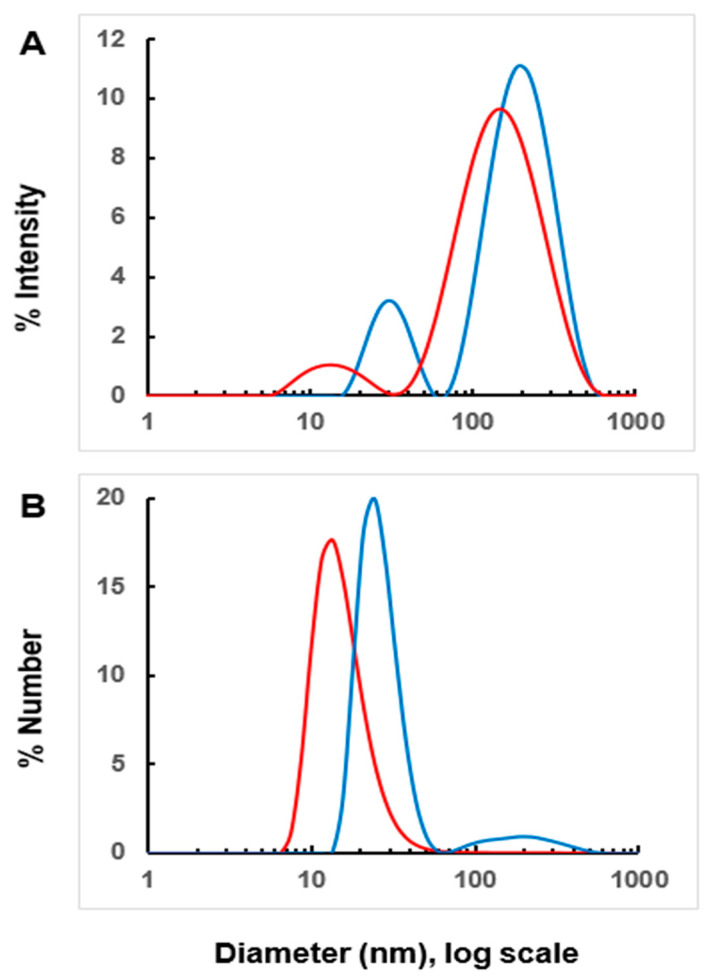
DLS analysis of 20K-SEC-Hep (blue line) and 106K-SEC-Hep (red line) EV samples. The size distribution is presented by intensity (**A**) and number (**B**).

The second aspect relates to the purity of the EV preparation. Figure 2 shows DLS spectra for 20K-SEC-Hep (blue line) and 106K-SEC-Hep (red line) EV samples. When presented by intensity (Figure 2A), the graphs show broad peaks that point to a polydispersity of samples. This is consistent with an expectation that both EV samples have multiple species. When presented by number (Figure 2B), there is a single peak in each sample. 20K-SEC-Hep EVs are represented by approximately 28 nm peaks, while 106K-SEC-Hep EVs are represented by approximately 15 nm peaks. Although they do not provide information about the purity of the EV samples, DLS data concur well with the EV sizes expected after 20,000× *g* and 106,000× *g* centrifugations. A further assessment of EV sample purity was made based on MRM quantification of several complement and EV proteins. C5b, C6, C7, C8, and C9 are common soluble proteins appearing in plasma at concentrations ranging from approximately 60 µg/mL to 120 µg/mL, which based on their molecular weights, cover approximately 0.6 µmol/L to 1.0 µmol/L. Another soluble complement protein C3 is present at approximately 10 times higher concentrations (1.2 mg/mL or 6.4 µmol/L). We have tracked the concentration of C3 in our EV preparations after every purification step to obtain an estimate of how pure the final 20K-SEC-Hep and 106K-SEC-Hep EVs are. Table 1 shows the concentrations of C3 in various EV preparations and a relative EV purity expressed in fold increase in comparison to the original plasma. The calculations were made based on 103 pmol/mg of total protein measured for C3 in the original plasma. For 20K-SEC-Hep and 106K-SEC-Hep EVs, we achieved approximately 1200-fold and 1500-fold purification, respectively. An additional observation is that starting with an approximately 10 times higher concentration of soluble C3 over soluble C5, C6, C7, C8, or C9 in the original plasma, we obtained EV samples with C3 concentrations lower than the concentrations of those proteins (Table 2).

To further characterize the final preparations of EVs, we have measured five proteins (TSG101, flotillin-1, EHD4, moesin, and integrin beta-1) that are typical for any EV preparation (Table 2). We also measured six plasma membrane-specific proteins and two endosomal membrane-specific proteins that are expected to appear mostly in 20K EVs and 106K EVs, respectively [23,24,25,26]. As expected, plasma membrane-specific proteins were found enriched in 20K-SEC-Hep EVs, while endosomal membrane-specific proteins were found enriched in 106K-SEC-Hep EVs.

Based on the data presented in Table 1 and Table 2, we concluded that the final EV preparations were well-purified and unlikely to be contaminated with soluble complement proteins, but rather show the presence of complement proteins associated with the EVs.

### 3.2. MAC Proteins in the EV Preparations

Table 2 shows the amounts of MAC proteins detected in 20K-SEC-Hep and 106K-SEC-Hep EVs. Based on these numbers, we have made two important conclusions.

First, the amounts of MAC proteins detected in 20K-SEC-Hep EVs were higher than the amounts of MAC proteins detected in 106K-SEC-Hep EVs (Table 2). C7 and C8 in the 106K-SEC-Hep EVs show a low quality and poor reproducibility of MRM signals and were marked as “not determined” (ND). It is possible that trace amounts of these proteins are present in the sample; however, their quantification is not possible under the conditions used. Overall, we have concluded that 20K-SEC-Hep EVs retain MAC proteins better than 106K-SEC-Hep EVs. This is an important conclusion since 20K-SEC-Hep EVs mainly represent the vesicles generated from the cell plasma membrane. In other words, 20K-SEC-Hep EVs are the vesicles in which MAC proteins are supposed to be, while the presence of MAC proteins in 106K-SEC-Hep EVs does not have a functional meaning and can be interpreted as a trace contamination.

Second, although all proteins that reconstitute MAC are present in 20K-SEC-Hep EVs, their stoichiometry is not consistent with the stoichiometry of preformed MAC. MAC is an irreversible complex that once formed, has a specific stoichiometry with 18 units of C9 and only one unit each of C5b, C6, C7, and C8. We found approximately equal amounts of every protein that constitutes MAC in 20K-SEC-Hep EVs (Table 2).

## 4. Conclusions

Taken together, this study shows that EVs from normal human plasma can carry individual MAC proteins, but there are no signs of the presence of preformed MAC. Presumably, plasma EVs can deliver individual MAC proteins to the targeted cells. The consequences of this delivery warrant further study.

## Figures and Tables

**Table 1:** Quantification of C3 in various EV preparations.

	20K	20K-SEC	20K-SEC-Hep	106K	106K-SEC	106K-SEC-Hep
C3/C3b *, pmol/mg total protein	14.8 ± 1.8	0.78 ± 0.12	0.087 ± 0.016	6.8 ± 0.8	0.59 ± 0.09	0.068 ± 0.015
Relative EV purity **, fold increase	7	132	1184	15	175	1515

* MRM measurements were performed in triplicate for three transitions per peptide and two peptides per C3 (*n* = 18). Data are presented as the mean ± SD. ** Plasma concentration of C3 was 103 pmol/mg total protein and was used to calculate a relative EVs purity as a fold increase based on concentrations of C3 in EV preparations.

**Table 2:** Quantification of MAC and EV proteins in 20K-SEC-Hep and 106K-SEC-Hep preparations.

Proteins	20K-SEC-Hep	106K-SEC-Hep
Complement		
C3/C3b	0.09 ± 0.02	0.07 ± 0.02
C5/C5b	1.4 ± 0.20	0.09 ± 0.02
C6	1.1 ± 0.18	0.07 ± 0.02
C7	0.8 ± 0.16	ND
C8/C8a	0.9 ± 0.16	ND
C8/C8b	0.8 ± 0.12	ND
C9	1.2 ± 0.18	0.09 ± 0.02
EVs generic		
TSG101	2.7 ± 0.8	1.0 ± 0.3
flotillin-1	3.7 ± 1.1	0.8 ± 0.3
EHD4	6.1 ± 1.5	11.6 ± 3.1
moesin	157 ± 29	13.3 ± 3.2
integrin beta-1	6.4 ± 1.8	1.0 ± 0.3
EVs plasma membrane-specific		
integrin alpha-IIb	132 ± 35	5.4 ± 1.3
integrin beta-3	127 ± 38	5.1 ± 1.3
platelet glycoprotein Ib alpha	19.1 ± 5.0	ND
platelet glycoprotein Ib beta	17.2 ± 4.1	ND
platelet glycoprotein V	17.7 ± 5.5	ND
platelet glycoprotein IX	18.2 ± 5.3	ND
EVs endosomal membrane-specific		
integrin alpha-2	10.8 ± 3.3	19.3 ± 6.0
cytochrome P-450 5A1	ND	1.1 ± 0.3

Data are shown in pmol of targeted protein per mg of total protein. MRM measurements were performed in triplicate for three transitions per peptide and two peptides per protein (*n* = 18). Data are presented as the mean ± SD. ND stands for not determined.

## Data Availability

All data presented in this study are available on request from the corresponding author.

## Supplementary Materials

The following supporting information can be downloaded at https://www.mdpi.com/article/10.3390/proteomes12030021/s1: This includes Figure S1 The amino acid sequences of COM1 and COM2 QconCATs. And Table S1 Transitions used for quantification.
