# Supplementary material for: Quantitative Analysis of Complement Membrane Attack Complex Proteins Associated with Extracellular Vesicles
Source: Proteomes. 2024 Jul 12;12(3):21. doi: 10.3390/proteomes12030021 (PMC11270256; doi:10.3390/proteomes12030021)
Supplement: Supplementary file 1 [file proteomes-12-00021-s001.zip › proteomes-3018004-supplementary.pdf]

Supplementary Materials

Quantitative analysis of complement membrane attack complex proteins associated with extracellular vesicles

Illarion V. Turko[1]

[1]Institute for Bioscience and Biotechnology Research, National Institute of Standards and Technology, University of Maryland, Rockville, Maryland 20850, USA

Figure S1. The amino acid sequences of COM1 and COM2 QconCATs. Peptides (marked red) with 6 amino acid residues flanking sequences on both sides (marked black) from proteins listed below were randomly concatenated in COM1 and COM2 sequences, respectively. 6 x His tag and linker sequence (marked orange) were from expression vector

COM1
Number of amino acids: 552
Molecular weight for ^{15}N-labeled: 64532
Theoretical pI: 9.23

MLGGEYRVLFYVDSEKLKQNDFNSVEEKKCKSSGWHFVVKFSSHGCDSTCERLYYG
DDEKYFRKPYNFLKYHFEALQRVKRLPLEYSYGEYRDLFRDFVIRNRDVVLTTTFVDDI
KALPTTYEKGEYFAFSFGFKIPGIFELGISSQSDRGKHYIRFTTKAKDLHLSDVFLKALN
HLPLEYNSALYSRIFDDFGKDFYRLSGNVLSYTFQVKINNDFNKLSEKHEGSFIQGAEK
SISLIRGGRSEYGAALAWEKGSSGLEEKTFSEWLESVKENPAVIHEVLRHTSLGPLEAK
RQNLRRIISRVRGGSSGWSGGLAQNRSTITYRKQNLERAIEDYINEFSVRKCHTCQIFR
SRSIEVFGQFNGKRCTDAVFLHARSDLEVAHYKLKPRSLNTRFRKPYNVESYTPQTQG
KYEFILKRGEGRAVNITSENLIDDVVSLIRGGTRKYAGNKRRYSAWAESVTNLPQVIKQ
KLTPLYELVKEVPCASGEKNFRTEHYEEQIEAFKSIIQEKAKNDFKIGGAIEEVYVSLGV
SVGKCRGILNKLAAALEHHHHHH

Complement component C6 (P13671, 22-934 aa)
1-2. FTTKAKDLHLSDVFLKALNHLPLEYNSALYSRIFDDFG (1185.6, 1860.0)
3-6.
NKLSEKHEGSFIQGAEKSISLIRGGRSEYGAALAWEKGSSGLEEKTFSEWLESVKENP
AVI (1201.6, 1223.6, 805.4, 1224.6)

Complement component C7 (P10643, 23-843 aa)
1. GKDFYRLSGNVLSYTFQVKINNDFN (1454.8)
2-4. LGGEYRVLFYVDSEKLKQNDFNSVEEKKCKSSGWHFVVKFSSHGC (1098.6, 1208.5, 1045.5)
5-6. AGNKRRYSAWAESVTNLPQVIKQKLTPLYELVKEVPCAS (1805.0, 1074.6)

Complement component C8 alpha chain (P07357, 31-584 aa)
1-2. DSTCERLYYGDDEKYFRKPYNFLKYHFEAL (1001.4, 908.5)
3. IISRVRGGSSGWSGGLAQNRSTITYR (1332.6)
4. IHEVLRHTSLGPLEAKRQNLRR (1051.6)

Complement components C8 beta chain (P07358, 55-591 aa)
1. LNTRFRKPYNVESYTPQTQGKYEFILK (1738.9)
2. FSFGFKIPGIFELGISSQSDRGKHYIR (1617.8)
3. VFLHARSDLEVAHYKLKPRSL (1060.5)
4. LQRVKRLPLEYSYGEYRDLFRDF (1388.7)
5. AKNDFKIGGAIEEVYVSLGVSVGKCRGILN (1776.0)

Complement component C9 (P02748, 22-559 aa)
1. QMFRSRSIEVFGQFNGKRCTDAV (1224.6)
2. GEKNFRTEHYEEQIEAFKSIIQEK (1522.7)
3-4. FVMRNRDVVLTTTFVDDIKALPTTYEKGEYFAF (1464.8, 921.5)
5. KRGEGRAVNITSENLIDDVVSLIRGGTRKY (1970.1)
6. KQNLERAIEDYINEFSVRKCHTCQ (1454.7)

COM2
Number of amino acids: 675
Molecular weight for ^{15}N-labeled: 76502
Theoretical pI: 8.30

MSSENKFQNSAILTIQPKQLPGGQEKGRNKFVTVQATFGTQVVEKVVLVSLEFNVKTD
APDLPEENQAREGYRAIEQTIKSGSDEVQVGQQRTFISPIAYAYKVSITSITVENVFVKY
KATLLRYFYNKVVTEADVYITFGIREDLKDDNGSFKENSQYQPIKLQGTLPVEARENSL
YLTAFTVIGIRKAFDICSFYLKIDTQDIEASHYRGYGNSDCLAFKVHQYFNVELIQPGAVK
VYAYYNEEDLKALVEGVDQLFTDYQIKDGHVILVQAERSGIPIVTSPYQIHFTKTPKYFK
AAFVKRAPSTWLTAYVVKVFSLAVTIPPKSSLSVPYVIVPLKTGLQEVEVKAAVYHHFIS
DGVRKSLKVVFIQTDKTIYTPGSTVLYRIFTVNHLGLARSNLDEDIIAEENIVSRSEFPES
YSVWKGGSASTWLTAFALRVLGQVNWTDNHKALLVGEHLNIIVTPKSPYIDKITHYNYLI
LSKGKIIHRSEETKENEGFTVTAEGKGQGTLSFIPSFRLVAYYTLIGASGQREVVADSW
VDVKDSCVGSFLYGKKVEGTAFVIFGIQDGEQRISLPESLKRIPIEDGSGEVVLSRKVLL
DGVQNPRAEDLVGDGVAKLSINTHPSQKPLSITVRTKKQELGRLHIKTLLPVSKPEIRSY
FPESKLAAALEHHHHHH

Complement C5 beta chain (P01031, 19-673 aa)
1. LSSENKFQNSAILTIQPKQLPGGQ (1358.8)
2. RYFYNKVVTEADVYITFGIREDLKDD (1581.8)
3. LEFNVKTDAPDLPEENQAREGYRAI (1454.7)
4-5. WTDNHKALLVGEHLNIIVTPKSPYIDKITHYNYLILSKGKIIHR (1616.0, 1363.8)

Complement C5 alpha chain (P01031), 678-1676 aa)
1. GRLHMKTLLPVSKPEIRSYFPES (1251.8)
2. SYSVWKGGSASTWLTAFALRVLGQVN (1436.7)
3-5. DNGSFKENSQYQPIKLQGTLPVEARENSLYLTAFTVIGIRKAFDIC (1105.5, 1082.6, 1695.9)
6. CSFYLKIDTQDIEASHYRGYGNSD (1446.7)
7. NEEDLKALVEGVDQLFTDYQIKDGHVIL (1837.9)
8. IAYAYKVSITSITVENVFVKYKATLL (1534.9)

Complement C3 beta-chain (P01024, 23-667 aa)
1. EKGRNKFVTVQATFGTQVVEKVVLVSL (1652.9)
2. FIQTDKTIYTPGSTVLYRIFTVNH (1369.7)
3-5.
FLYGKKVEGTAFVIFGIQDGEQRISLPESLKRIPIEDGSGEVVLSRKVLLDGVQNPRAED LVG (1864.9, 1469.8, 1109.6)
6. MVQAERSGIPIVTSPYQIHFTKTPKYFK (1787.0)
7. GDGVAKLSINTHPSQKPLSITVRTKKQEL (1890.1)
8-9. FIPSFRLVAYYTLIGASGQREVVADSWVDVKDSCVGS (1510.8, 1344.7)

Complement C3 alpha-chain (P01024, 672-1663 aa)
1. HLGLARSNLDEDIIAEENIVSRSEFPES (1815.9)
2-4. VTIPPKSSLSVPYVIVPLKTGLQEVEVKAAVYHHFISDGVRKSLKVV (1400.8, 1001.5, 1470.7)
5. AAFVKRAPSTWLTAYVVKVFSLAV (1334.7)
6. RSEETKENEGFTVTAEGKGQGTLS (1280.6)
7. DCLAFKVHQYFNVELIQPGAVKVYAYYN (1841.0)
8. IEQTIKSGSDEVQVGQQRTFISPI (1288.6)

Table S1. Transitions used for quantification.

Protein	Peptide		Precursor (m/z)	Product Ions (m/z)		
				1	2	3
C3/C3b	IPIEDGSGEVVLSR	L	735.9	759.4 (y7)	846.5 (y8)	903.5 (y9)
		H	744.4	769.4 (y7)	857.4 (y8)	915.5 (y9)
	VLLDGVQVPR	L	548.3	598.4 (y5)	655.4 (y6)	770.4 (y7)
		H	555.3	607.3 (y5)	665.4 (y6)	781.4 (y7)
C5/C5b	TDAPDLPEENQAR	L	728.3	843.4 (y7)	956.5 (y8)	1168.6 (y9)
		H	737.3	855.4 (y7)	969.4 (y8)	1183.5 (y9)
	FQNSAILTIQPK	L	680.4	699,4 (y6)	812.5 (y7)	883.6 (y8)

		H	688.4	707.4 (y6)	821.5 (y7)	893.5 (y8)
C6	TFSEWLESVK	L	613.3	761.4 (y6)	890.5 (y7)	977.5 (y8)
		H	619.3	769.4 (y6)	899.4 (y7)	987.5 (y8)
	SEYGAALAWEK	L	612.8	646.4 (y5)	717.4 (y6)	788.4 (y7)
		H	619.3	653.3 (y5)	725.4 (y6)	797.4 (y7)
C7	VLFYVDSEK	L	550.3	577.3 (y5)	740.3 (y6)	887.4 (y7)
		H	555.3	583.3 (y5)	747.3 (y6)	895.4 (y7)
	QNDFNSVEEK	L	605.3	705.3 (y6)	852.4 (y7)	967.4 (y8)
		H	612.3	713.3 (y6)	861.4 (y7)	977.4 (y8)
C8/C8a	HTSLGPLEAK	L	526.8	557.3 (y5)	614.4 (y6)	727.4 (y7)
		H	533.3	563.3 (y5)	621.4 (y6)	735.4 (y7)
	LYYGDDEK	L	501.7	506.2 (y4)	563.2 (y5)	726.3 (y6)
		H	506.2	511.2 (y4)	569.2 (y5)	733.3 (y6)
C8/C8b	SDLEVAHYK	L	531.3	617.3 (y5)	746.4 (y6)	859.5 (y7)
		H	537.2	625.3 (y5)	755.4 (y6)	869.4 (y7)
	IPGIFELGISSQSDR	L	809.9	849.4 (y8)	962.5 (y9)	1091.5 (y10)
		H	819.4	861.4 (y8)	975.5 (y9)	1105.5 (y10)
C9	ALPTTYEK	L	461.7	540.3 (y4)	641.3 (y5)	738.4 (y6)
		H	466.2	545.3 (y4)	647.3 (y5)	745.3 (y6)
	DVVLTTTFVDDIK	L	733.4	736.4 (y6)	837.4 (y7)	938.5 (y8)
		H	740.4	743.4 (y6)	845.4 (y7)	947.5 (y8)
TSG101	GVIDLDVFLK	L	559.8	621.4 (y5)	734.4 (y6)	849.5 (y7)
		H	565.3	627.3 (y5)	741.4 (y6)	857.4 (y7)
	IYLPYLHEWK	L	681.4	712.4 (y5)	875.4 (y6)	972.5 (y7)
		H	688.3	721.4 (y5)	885.4 (y6)	983.5 (y7)
flotillin-1	VTGEVLDILTR	L	608.4	617.4 (y5)	730.4 (y6)	829.5 (y7)
		H	615.3	625.3 (y5)	739.4 (y6)	839.5 (y7)
	AQADLAYQLQVAK	L	709.9	849.5 (y7)	920.5 (y8)	1033.6 (y9)
		H	718.4	859.5 (y7)	931.5 (y8)	1045.6 (y9)
EHD4	SISVIDSPGILSGEK	L	751.4	800.5 (y8)	887.5 (y9)	1002.5 (y10)
		H	759.4	809.4 (y8)	897.5 (y9)	1013.5 (y10)
	LDISDEFSEAIK	L	683.8	547.3 (y5)	694.4 (y6)	823.4 (y7)
		H	690.3	553.3 (y5)	701.4 (y6)	831.4 (y7)
moesin	IGFPWSEIR	L	552.8	690.4 (y5)	787.4 (y6)	991.5 (y8)
		H	559.3	699.3 (y5)	797.4 (y6)	1003.5 (y8)
	APDFVFYAPR	L	591.8	653.3 (y5)	752.4 (y6)	899.5 (y7)
		H	598.3	661.3 (y5)	761.4 (y6)	909.4 (y7)
Integrin beta-1	IGGGSFVEK	L	492.3	522.2 (y4)	609.3 (y5)	666.3 (y6)
		H	497.2	527.3 (y4)	615.3 (y5)	673.3 (y6)
	GEVFNELVGK	L	546.3	659.4 (y6)	806.4 (y7)	905.5 (y8)
		H	552.3	667.3 (y6)	815.4 (y7)	915.5 (y8)
Integrin alpha-IIb	FGSAIAPLGDLDR	L	666.4	688.4 (y6)	785.4 (y7)	856.5 (y8)
		H	674.3	697.3 (y6)	795.4 (y7)	867.4 (y8)
	GEAQVWTQLLR	L	650.9	816.5 (y6)	915.5 (y7)	1043.6 (y8)
		H	659.3	827.4 (y6)	927.5 (y7)	1057.6 (y8)
Integrin beta-3	VLEDRPLSDK	L	586.3	559.3 (y5)	715.4 (y6)	830.4 (y7)
		H	593.3	565.3 (y5)	725.4 (y6)	841.4 (y7)
	GSGDSSQVTQVSPQR	L	766.9	817.4 (y7)	914.5 (y8)	1042.6 (y9)
		H	777.3	827.4 (y7)	927.5 (y8)	1057.5 (y9)
GP Ib alpha	GLGELQELYLK	L	631.9	665.4 (y5)	793.4 (y6)	906.5 (y7)
		H	638.3	671.4 (y5)	801.4 (y6)	915.5 (y7)
	GVLQGHLESSR	L	591.8	728.4 (y6)	785.4 (y7)	913.4 (y8)
		H	600.3	739.3 (y6)	797.4 (y7)	927.4 (y8)
GP Ib beta	LSLTDPLVAER	L	607.3	684.4 (y6)	799.4 (y7)	900.5 (y8)
		H	614.3	693.4 (y6)	809.4 (y7)	911.4 (y8)

	LLPYLAEDELR	L	666.4	732.4 (y6)	845.4 (y7)	1008.5 (y8)
		H	673.3	741.3 (y6)	855.4 (y7)	1019.5 (y8)
GP V	SIAPGAFDR	L	467.2	508.3 (y4)	565.3 (y5)	662.3 (y6)
		H	473.2	515.2 (y4)	573.2 (y5)	671.3 (y6)
	LPNLSSLTLSR	L	600.9	676.4 (y6)	763.4 (y7)	876.5 (y8)
		H	608.3	685.4 (Y6)	773.4 (y7)	887.5 (y8)
GP IX	GHGLTALPALPAR	L	637.4	737.5 (y7)	808.5 (y8)	909.6 (y9)
		H	646.3	747.4 (y7)	819.5 (y8)	921.5 (y9)
	TPEALLQVR	L	513.8	628.4 (y5)	699.5 (y6)	828.5 (y7)
		H	520.3	637.4 (y5)	709.4 (y6)	839.5 (y7)
Integrin alpha-2	FVQGLDIGPTK	L	587.8	630.3 (y6)	743.4 (y7)	700.4 (y8)
		H	594.3	637.3 (y6)	751.4 (y7)	809.4 (y8)
	IGQTSSSVSFK	L	570.8	654.3 (y6)	741.4 (y7)	842.4 (y8)
		H	577.3	661.3 (y6)	749.4 (y7)	851.4 (y8)
CYP 5A1	WYSTSAFSR	L	552.8	567.3 (y5)	668.3 (y6)	755.4 (y7)
		H	559.2	575.3 (y5)	677.3 (y6)	765.3 (y7)
	DELNGFFNK	L	542.3	555.3 (y4)	612.3 (y5)	726.4 (y6)
		H	548.2	561.3 (y4)	619.3 (y5)	735.3 (y6)

Transitions are listed for both unlabeled, light (L) and fully ^{15}N-labeled, heavy (H) peptides. All precursors were +2 charge and product ions were +1 charge. Additionally, y-ion information is included for reference.
